# Virtual and fiber-optic bronchoscopy in patients with indication for tracheobronchial evaluation

**DOI:** 10.4103/1817-1737.62474

**Published:** 2010

**Authors:** Fulya Adali, Atilla Uysal, Sibel Bayramoglu, Nurten Turan Guner, Gulizar Yilmaz, Tan Cimilli

**Affiliations:** *Department of Radiology, Bakirkoy Dr. Sadi Konuk Training and Research Hospital, Istanbul, Turkey*; 1 *Department of Pulmonary Medicine, Yedikule Chest Medicine and Chest Surgery Training and Research Hospital, Istanbul, Turkey*

**Keywords:** Fiber-optic bronchoscopy, multislice computed tomography, tracheobronchial pathologies, virtual bronchoscopy

## Abstract

**OBJECTIVE:**

The aim of this study was to compare the results of virtual bronchoscopy (VB) images in defining tracheobronchial pathologies with those of fiber-optic bronchoscopy (FOB) in patients with clinical indication for bronchoscopy.

**METHODS:**

Twenty-two patients with bronchoscopy indication were evaluated with FOB and VB. The VB results were evaluated blindly, independent of the FOB results.

**RESULTS:**

In 19 of the 22 patients, tracheobronchial abnormalities were present on FOB, whereas 3 patients had normal findings on FOB. In 17 of 19 patients, VB demonstrated the FOB diagnosis of tracheobronchial abnormality. While FOB detected 11 endoluminal lesions, VB detected 6. While FOB detected 20 obstructive lesions, VB detected 26. In evaluating external compression, FOB detected 2 lesions and VB detected 15.

**CONCLUSIONS:**

VB is a non-invasive, uncomplicated, and reproducible examination method in patients with an indication for thorax examination. Virtual bronchoscopy could find a clinically broader field of application in the future.

Virtual bronchoscopy (VB) is a three-dimensional reconstruction that shows a real-like anatomical view of the tracheobronchial tree. Although the technique was described in the mid 1990s, it is generating new interest as a result of improvements in computer hardware and software and advances in multislice computed tomography (MSCT) scanner technology that allow the acquisition of isotropic data.[1,2]

Fiber-optic bronchoscopy (FOB) is considered the ‘gold standard’ for the detection and diagnosis of tracheobronchial pathology because it permits direct visualization of the airway lumen. However, it has potentially hazardous complications in severely ill patients.[3] Virtual bronchoscopy allows the non-invasive evaluation of endoluminal lesions and airway stenoses. In addition, cranial morphology may be evaluated in a similar way to FOB. The only limitation of the method is that, with the current technology, mucosal details cannot be evaluated and a biopsy cannot be performed. Although FOB enables direct visualization of the bronchial mucosa, the operator is unable to see beyond an obstructive lesion and not all patients may tolerate the procedure.[4]

We aimed to compare the results of VB images obtained via MSCT and multiplanar reconstruction (MPR) data in defining tracheobronchial pathologies with those of FOB in patients with clinical indication for bronchoscopy.

## Materials and Methods

This prospective study was performed between March 2006 and January 2007. The subjects included in this study were patients with clinical indications for bronchoscopy. The clinical indications for performing FOB included chronic cough, hemoptysis, persisting or recurrent pulmonary infections (cough, fever, sputum), suspicion of foreign-body aspiration, abnormal findings at physical examination (abnormal breathing sounds, stridor, dyspnea) and chest X-ray abnormality. Chest X-ray was used in choosing the subjects. The patients who had abnormalities on their chest X-ray were referred for chest thorax computed tomography (CT) for further investigation.

The study included 22 patients who underwent thorax CT and VB with MSCT in Yedikule Hospital for Chest Disease and Thoracic Surgery and Dr. Sadi Konuk Training and Research Hospital. Virtual bronchoscopy results were evaluated blindly and independently from those of FOB, which was performed at a different time. Fiber-optic bronchoscopy was performed within 24 h of the thorax CT examination. The local ethical committee had approved the study protocol and the patients had given informed consent before the examinations.

During the VB examination, performed using a Somatom Sensation 40 (Siemens Medical Systems, Forchheim, Germany), the tracheobronchial tree was screened axially with 0.6 mm slices from the thoracic inlet to the end of the diaphragm after administering intravenous contrast material. The pitch factor used in the Lung Care Program was maintained at a constant 1.4 at 120 kV, and screenings were performed at 100 mAS on average, despite some changes on an individual basis. Volumetric screening was completed in approximately 10 s during a single breath. Axial CT, coronal and sagittal MPR images with a slice thickness of 1 mm and with section intervals of 1 mm were reconstructed, and virtual bronchoscopic images obtained via the 3D Shaded Surface Display (SSD) computer program were evaluated simultaneously, dividing the computer screen into four equal quadrants in multi-view mode. Two-dimensional views were evaluated at the standard parenchymal window (level -600, wideness 1200) and mediastinal window (level -40, wideness 400) for lesions’ endobronchial extensions, relations with neighboring structures and accompanying pathologies. The lumen of the tracheobronchial tree was evaluated by moving from the proximal trachea using the ‘syngo Fly Through’ program.

The tracheobronchial tree was investigated after dividing it into 28 segments for an objective comparison between VB and FOB. Endoluminal and obstructive pathologies were grouped according to Finkelstein and coworkers’ classification as ‘endoluminal lesions’ leading to <50% narrowing in the lumen (mild–moderate stenoses) and as ‘obstructive lesions’ leading to >50% narrowing (severe stenoses).[5] Moreover, external compressions detected via VB and MPR findings were recorded.

Fiber-optic bronchoscopy was performed by same pulmonologists, no later than 24 h after VB. Bronchoscopies were performed via the oral or nasal route with a bronchoscope (Olympus BF-30) under anesthesia (Lidocaine HCl 10 mg/dose spray – midazolam 5 mg IV). Lesions of the tracheobronchial tree were grouped as ‘endoluminal lesions’ and ‘obstructive lesions’ as with VB. The presence of mucosal lesions and external compression was also recorded. During FOB, samples were obtained using biopsy forceps, needle aspiration (Wang^©^, Gauge 119), brushing, bronchoalveolar lavage or a few of them. A specialized pathologist evaluated the materials. Final diagnoses were approved by histopathological analysis of specimens obtained during bronchoscopy.

### Statistical analysis

The FOB results were used as a reference for comparison with the VB results. Qualitative results regarding the description of tracheobronchial abnormalities with VB were defined as true-positive, true-negative, false-positive and false-negative findings. A true-positive finding was classified as an abnormality noted in both image modalities (FOB and VB), whereas a true-negative finding was defined as an absence of airway abnormalities on FOB and VB. A false-positive finding was defined as a tracheobronchial section noted as abnormal on VB but normal on FOB. A false-negative finding was defined as a tracheobronchial section that appeared normal on VB but abnormal on FOB. Sensitivity, specificity, positive predictive value (PPV), negative predictive value (NPV) and accuracy were calculated as follows: sensitivity: true-positive/false-negative + true-positive; specificity: true-negative/false-positive + true-negative; PPV: true-positive/true-positive + false-positive; NPV: true-negative/true-negative + false-negative; accuracy (%) = 100 × (true-positive + true-negative/true-positive + false-positive + true-negative + false-negative).

## Results

Twenty-two patients (6 female, 27.3%; 16 male, 72.7%) were enrolled in the study. Their mean age was 52 ± 22 years. The histopathological diagnoses and the numbers of each are listed in Table 1.

**Table 1:** The histopathological diagnosis

Diagnosis	Number of cases
Small cell cancer	6
Squamous cell cancer	5
Adeno cancer	1
Carcinoid tumor	1
Lymphoma	1
Granuloma	1
Pneumonia	1
Chronic inflammation	1
Endobronchial tuberculosis	2
Foreign body aspiration	1
Mucoid plug	1
Insufficient material	1
Total	22

**Figure 1:**
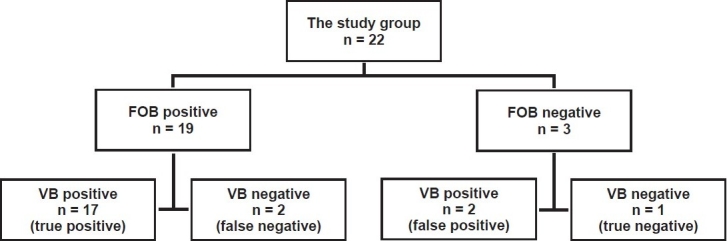
Flow diagram of fiber-optic bronchoscopy and virtual bronchoscopy results

In 19 (86%) of the 22 patients, tracheobronchial abnormalities were present on FOB, whereas 3 patients had normal findings on FOB. In 17 (89%) of these 19 patients, VB confirmed the tracheobronchial abnormality diagnosed by FOB. Two patients with findings of tracheobronchial abnormality on FOB had normal findings on VB. One patient had normal findings on both VB and FOB. Two patients with normal tracheobronchial findings on FOB had a tracheobronchial abnormality according to VB [Figure 1]. Based on these numbers, sensitivity and specificity were calculated as 89 and 33%, respectively, whereas PPV and NPV were 89 and 33%, respectively. Accuracy was 81%. According to the final diagnosis made based on histopathological findings and post-treatment observations, FOB had a sensitivity of 100% and specificity of 66.67%, PPV of 95.0% and NPV of 100%.

A total of 18 segments in each case were included in the evaluation. While FOB detected 11 endoluminal lesions, VB detected 6. Moreover, while FOB detected 20 obstructive lesions, VB detected 26. In evaluating external compression, FOB detected 2 lesions and VB detected 15. In evaluating mucosal pathologies (irregularities, hyperemia, edema, fragility), while VB did not provide any information, FOB detected 10 mucosal pathologies [Table 2].

**Table 2:** Number of lesions detected with FOB and VB

	FOB	VB
Endoluminal lesion	11	6
Obstructive lesion	20	26
External compression	2	15
Mucosal findings	10	0

VB = Virtual bronchoscopy, FOB = Fiber-optic bronchoscopy

## Discussion

The appropriate management of airway disease requires an accurate diagnosis. For these invasive procedures are usually required, such as conventional flexible endoscopy, which is not without risk to the patient.[6] Virtual bronchoscopy makes it possible to navigate the airways and to determine extrabronchial abnormalities.[7] Virtual bronchoscopic trials were initiated in the mid 1990s by Vining *et al*. that included a small number of patients.[2,8] In VB, intraluminal 3D views of the bronchi are formed by coding surface voxels based on central airways density differences accompanying 2D reconstructed views.[2,8,9] There are basically two different approaches in reconstructing VB views: SSD and volume rendering.[10] In our study, SSD was used to create intraluminal views of the bronchi.

Currently, FOB is the gold standard in endobronchial evaluation of the tracheobronchial tree. In most studies, FOB was used as the reference ‘gold standard’ for comparison between FOB and VB.[11] In our study, the reference ‘gold standard’ was FOB. To provide a standard of reference, the pulmonologist who performed the FOB was blinded to the results of the VB.

In the present study, VB detected tracheobronchial lesions with a sensitivity of 89% and an overall accuracy of 81%, as in previous studies.[12,13] Moreover, VB detected endoluminal lesions as effectively as FOB, except for five locations in two cases. In the retrospective assessment of these two cases detected by FOB but not by VB, the result of VB did not change and the difference was thought to be due to the size of the lesions. The sensitivity of VB in detecting endoluminal lesions was related to the size of the lesions. Virtual bronchoscopy may detect endoluminal pathologies >5 mm easily. In a previous study, it was reported that the sensitivity and specificity of FOB were 47–88% and 58–90%, respectively, for lesions of 3 to 10 mm in size and its sensitivity increased when only lesions larger than 5 mm in diameter were considered.[7,13,14]

**Figure 2:**
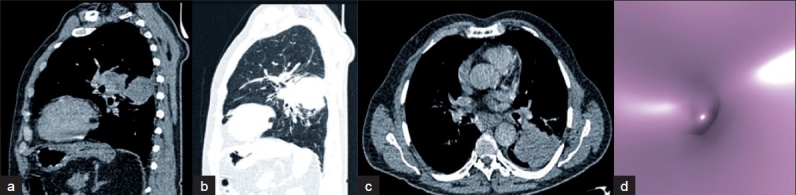
Squamous cell carcinoma case a, b, c - Sagittal MPR and axial views of the stenosis in the distal left lower superior segment, d - Complete obstruction in VB at the same location. This case was reported as normal by FOB because it did not reach distal regions.

Virtual bronchoscopy detected obstructive lesions as effectively as FOB. In addition, it detected obstructive lesions of subsegmental bronchial branches where FOB could not reach, as in one patient in our study group [Figure 2]. Moreover, VB could be performed in regions distal to stenoses, where FOB was not possible. Because of this advantage, we think that VB might be used to complement FOB in selected cases. In the literature, VB was found to be superior to FOB in the evaluation of regions beyond stenoses and in grading stenoses.[15,16]

With an increase in the degree of obstruction, detection of lesions became easier with both methods. However, with FOB it was difficult to differentiate severe stenoses from obstructions.[15] Virtual bronchoscopy offered the advantage of being able to visualize areas beyond even high-grade stenoses. In our study, with VB, we were able to pass through regions of severe stenoses according to FOB, to the open airway beyond.

Fiber-optic bronchoscopy cannot show the situation in regions distal to severe stenoses or total occlusion, the extension of the tumor, or whether a collapse has occurred or not. Virtual bronchoscopy, on the other hand, may be used to assess the bronchus distal to the stenoses, the length of the stenoses and their positional location.[9,10,14] These details are important for endobronchial procedures such as laser therapy, stent placement and transbronchial radiotherapy.[17–19] Fleiter and colleagues stated that VB could be used in post-endobronchial therapy follow-up.[15]

**Figure 3:**
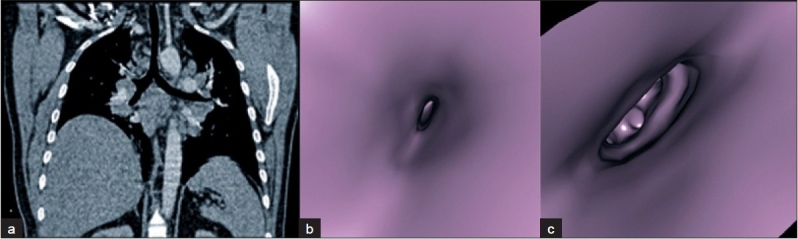
Lymphoma case a - Coronal MPR view of the external compression before the right middle lobe medial–lateral division. b, c - A view of the external compression at the right middle lobe proximal to medial–lateral segment division and lingual segment. Fiber-optic bronchoscopy reported in this case as an enlargement in the right upper intermediate and left upper–lower lobe carinae, severe stenosis in the right main bronchus, right middle lobe, lower lobe, and lingula, and moderate stenosis in the left lower lobe

**Figure 4:**
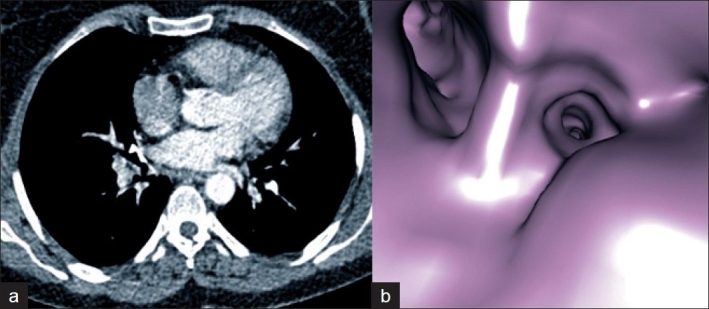
Chronic inflammation case a, b - Axial CT and VB images of the external compression due to calcified lymphadenopathy on the antero-medial wall at the entrance of the right middle lobe. This case was reported by FOB as an obstruction in the right medial middle lobe

While FOB is difficult to use in evaluating external compressions, VB with the help of MPR may provide comprehensive information. In our study, while FOB detected 2 external compressions, VB detected 15. In our only lymphoma case, while FOB could not clarify the etiology of various degrees of obstructions, VB could have detected, with the help of MPR views, that asymmetrical branching in more detailed anatomical locations was due to external compressions caused by lymphadenopathy [Figure 3]. In the case where histopathologic findings indicated a chronic inflammation and VB detected asymmetrical stenoses in the right middle lobe medial segment, in the evaluation with MPR views it was determined to be an external compression due to calcified lymphadenopathy. With FOB, this lesion had been reported as an obstruction in the same location and its etiology could not be clarified [Figure 4]. Virtual bronchoscopy allows quantitative measurements like the size of a lesion, its relationship with neighboring structures, the length of a stenosis and the diameter of a bronchus, via multiplanar images. Therefore, in the evaluation of patients for surgery, in contrast to FOB, VB and MPR should be considered together.

Foreign body aspiration is a common and serious cause of respiratory difficulties in children.[20] Many cases of foreign body aspiration may not be recognized initially. Virtual bronchoscopy can be especially valuable for evaluation of suspected tracheobronchial stenosis and foreign body aspiration in children,[1] as in one patient in our study group. Furthermore, VB can be easily used for identification of congenital variants such as tracheal and bronchial diverticula.[21]

Post-intubation tracheal stenosis remains the most frequent indication for tracheal surgery. Rigid bronchoscopy has traditionally been considered the technique of choice for the preoperative diagnostic assessment (tracheal resection and reconstruction). Taha *et al.*[22] suggest that VB and spiral CT scan with MPR may be considered as a substitute to direct endoscopic examination and the additional information on laryngeal function can be easily obtained during flexible nasolaryngoscopic examination of the awake patient. This policy can minimize patient morbidity and spare them an extra-anesthetic for evaluation. In cases with post intubation tracheal stenosis, VB may be considered a complementary technique in the preoperative assessment of laryngeal function and during follow-up.

In this study, intravenous contrast agent was administered in all patients. During the VB examination performed using MSCT to assess tracheobronchial pathologies, intravenous contrast agent may or may not be needed, depending on the clinical indication. For example, for imaging of suspected tracheobronchial stenosis, no intravenous contrast agent is needed. If, however, the indication is evaluation of airway involvement (especially, extraluminal compression) by malignant disease or suspected cases of a vascular ring or sling, intravenous contrast agent is essential.[1] For example, in our study, a biopsy was considered from a smooth surfaced, violet-colored nodular bulging detected by FOB, but due to its smooth contour, MSCT results were awaited. Virtual bronchoscopy together with MPR showed that the smooth surfaced bulging at the same level was the azygos vein. In these patients, VB combined with MPR provides useful guidance for the clinician before FOB, shortens the duration of FOB and prevents complications that could be secondary to the procedure, and in some cases it even obviates FOB.[18] FOB, with guidance from VB, especially avoids vascular structures and because anatomical details are known beforehand, VB helps ensure that FOB is safer and more successful.

Every procedure has advantages and disadvantages. Fiber-optic bronchoscopy allows direct evaluation of endoluminal and mucosal lesions and can guide biopsies for histological analysis. It also provides better assessment of mild degrees of stenosis. However, FOB has some limitation. It cannot pass through severely narrowed airways. It provides scarce information concerning the extent of extraluminal disease and it cannot be tolerated by some patients.[3] Virtual bronchoscopy is particularly useful for orientation within the tracheobronchial tree, and permits differentiation between intraluminal lesion and extraluminal airway compression. Virtual bronchoscopy allows one to assess the distal lesions and see beyond a stenosis. It may be able to distinguish between high-grade stenosis and complete occlusion.

Virtual bronchoscopy has some disadvantages, namely it cannot be used to evaluate mucosal details (fragility, color changes, vascularity), to show mucosal or submucosal extensions, or to perform a biopsy, and also this method cannot be used to depict functional stenoses due to tracheobronchomalasia.[12] The other limitation of VB was that it cannot be used to obtain a histological diagnosis (mucus plug, foreign body, embolism, benign mas and carcinoma).[23] Despite these disadvantages, improved resolution and a probable increase in diagnostic abilities are expected due to technological developments (aerosolized contrast materials and spectroscopic examination). Finally, FOB and VB are not two competing methods that can be compared regarding diagnostic accuracy. Fiber-optic bronchoscopy provides visualization and pathological specimens, whereas VB provides only visualization with a wider scope than FOB. Thus, FOB and VB only can be complementary to each other. Virtual bronchoscopy will never completely replace FOB for evaluation of lesions in the respiratory tract.

This study has several limitations. A possible limitation of our study was that we did not perform a stratification of the subjects regarding localizations of thoracic lesions. Additional limitation of this study is the small sample size.

In conclusion, VB is a non-invasive, uncomplicated and reproducible examination method, and in patients with an indication for thorax examination it may be performed without the additional risk of radiation. Furthermore, VB may be used in the evaluation of anatomical relationships in the thorax and extraluminal pathologies, in teaching about the structure-function relationship and bronchoscopy, and in patients refusing FOB. At the present time, we believe that VB might be complementary to FOB in selected cases. Virtual bronchoscopy may find a clinically broader field of application in the future.
